# Antenatal thyroid hormone therapy and antithyroid drug use in Norway from 2004 to 2018

**DOI:** 10.1530/EC-21-0631

**Published:** 2022-03-23

**Authors:** Kjersti S Bakken, Kristina Randjelovic Nermo, Bjørn Gunnar Nedrebø, Tim I M Korevaar, Tor A Strand

**Affiliations:** 1 Women’s Clinic, Innlandet Hospital Trust, Lillehammer, Norway; 2 Center for International Health, University of Bergen, Bergen, Norway; 3 Department of Microbiology, Innlandet Hospital Trust, Lillehammer, Norway; 4 Department of Medicine, Haugesund Hospital, Haugesund, Norway; 5 Department of Clinical Science, University of Bergen, Bergen, Norway; 6 Academic Center for Thyroid Diseases, Erasmus University Medical Center, Rotterdam, the Netherlands; 7 Department of Research, Innlandet Hospital Trust, Lillehammer, Norway

**Keywords:** thyroid dysfunction, pregnancy, Norway, thyroid therapy, MBRN, NorPD

## Abstract

**Objective:**

Thyroid disease during pregnancy is associated with adverse pregnancy outcomes and suboptimal fetal development. During the last decades, guidelines for diagnosing thyroid disease during pregnancy have changed considerably and there has been increased awareness. This study aimed to describe the prevalence of thyroid disease treatment over time among pregnant women in Norway.

**Design:**

Nationwide register-based study.

**Methods:**

We combined historical data from the Medical Birth Registry of Norway and the Norwegian Prescription Database, identifying pregnant women using thyroid therapy from 2004 to 2018.

**Results:**

A total of 855,067 pregnancies were included in the analyses. The proportion of women using thyroid hormone replacement therapy during pregnancy increased from 1.46% (*n*  = 800) in 2004 to 3.57% (*n*  = 1940) in 2018. The proportion of women using antithyroid medications also increased from 0.04% (*n*  = 20) in 2004 to 0.10% (*n*  = 56). During these 15 years, the mean maternal age increased by 0.9 years. When adjusting for age, the risk for being on thyroid hormone replacement therapy during pregnancy increased by an average of 5% per year (odds ratio: 1.05, 95% CI: 1.05–1.05).

**Conclusion:**

During the recent 15 years, there has been a substantial increase in the use of thyroid hormone therapy in Norwegian pregnant women. We speculate that this could be due to an increased awareness in combination with overdiagnosis because of inappropriate diagnostic criteria. To truly understand the possible causes and consequences of this development, further research is warranted.

## Background

Optimal thyroid function during pregnancy is essential for both the mother and the developing fetus since thyroid hormones regulate the metabolism, growth, maintenance of pregnancy and development of the fetal CNS (1). During pregnancy, there is an increase in the size of the thyroid gland, the production of thyroid hormones, as well as iodine requirement (2). Maternal thyroid disease during pregnancy is associated with adverse outcomes depending on the type of dysfunction. Both overt hypothyroidism, as well as Graves’ hyperthyroidism, is associated with miscarriage, pre-eclampsia, preterm birth and low birth weight, and suboptimal offspring neurocognitive development (2). In the last two decades, some but not all studies have shown that milder forms of thyroid dysfunction such as subclinical hypothyroidism and isolated hypothyroxinemia are associated with a higher risk of subfertility and adverse pregnancy outcomes (3). Publication of national and international guidelines since 2007 have put more emphasis on the relevance of preconception thyroid function for women with subfertility, as well as gestational thyroid dysfunction, which has led to increased awareness among physicians and case-based or even universal screening efforts (2). It remains unknown if levothyroxine therapy for mild forms of thyroid hypofunction has any beneficial effects. Yet, recent insights have suggested that the definition of thyroid dysfunction in guidelines published before 2017 has caused overdiagnosis of (subclinical) hypothyroidism and subsequent overtreatment (4, 5, 6). However, there is a lack of data that quantify this potential increase in thyroid hormone therapy from a national perspective. Interestingly, the diagnostic criteria and indications for antithyroid drug treatments for Graves’ hyperthyroidism have remained essentially unchanged. A Norwegian government report from 1999 raised concerns regarding thyroid function in pregnancy and highlighted the need for data on gestational thyroid disease. Despite this recommendation, population-based data on thyroid function in pregnancy has not yet been produced, and population-based antenatal screening is not recommended in Norway (7).

Iodine deficiency leads to inadequate thyroid hormone production and is considered to be the most important cause of preventable mental impairment (8, 9). While the fetus expresses thyroid hormone receptors as early as 9 weeks, it does not develop a functioning thyroid gland before 18–20 weeks and is therefore dependent on maternal thyroid hormones (10, 11). Although Norway has been considered iodine sufficient, pregnancy is a state of increased iodine demand and recent evidence suggests that mild and moderate iodine deficiency is prevalent and increasing among women of reproductive age (12). Part of this could be explained by the fact that the consumption of iodine-rich foods such as milk, yoghurt, and lean fish in Norway has declined over the past decades (12).

The aim of this study was to use national medical registers to describe the proportion of pregnant women using thyroid therapy during the last two decades in Norway.

## Material and methods

This is an observational population-based study combining historical data from the Medical Birth Registry of Norway (MBRN) and Norwegian Prescription Database (NorPD). MBRN is a nationwide health registry with information on all births in Norway, including pregnancies that ended or were terminated after week 12. The notification to MBRN contains personal identity number of the child and parents, maternal health information before and during pregnancy, any complications during birth or pregnancy, medicine use during pregnancy, birth interventions, birth complication, maternal complication, diagnoses in the child or congenital abnormalities. Additional information such as mother´s occupation, smoking, alcohol habits and information about assisted conception is only registered if the mother consents (https://www.fhi.no/en/hn/health-registries/medical-birth-registry-of-norway/medical-birth-registry-of-norway/). NorPD is a nationwide prescription registry established in January 2004 and contains information on all prescriptions dispensed to a person with a valid National identification number. All pharmacies in Norway are obliged to send data electronically on all prescribed drugs dispensed to individuals in ambulatory care (13).

NorPD provided information on women’s use of medication, limited to The Anatomical Therapeutic Chemical (ATC) code H03 (thyroid hormone replacement preparations and antithyroid preparations), and the date of dispensation. MBRN provided information on expected date of delivery (ultrasound due date) which was used to define whether or not the women used the medication within 1 year before pregnancy, during pregnancy or within 1 year after pregnancy. MBRN also provided information on maternal age in years at birth. The study includes women giving birth during the period 2004–2018.

The datafiles were deidentified by the Norwegian Institute of Public Health and the research team was provided with two separate files which were combined using the pseudonymized patient identification numbers.

Thyroid treatment was coded into three categories based on the ATC-codes: (i) thyroid hormone replacement therapy, which included women that had been given medication with the ATC-codes H03AA01 (levothyroxine sodium), H03AA02 (liothyronine sodium), H03AA03 (combinations of levothyroxine and liothyronine), and H03AA05 (thyroid gland preparations) during study period; (ii) antithyroid medication, which included women that had been given medication with the ATC-codes H03BA02 (propylthiouracil) and H03BB01 (carbimazole), during study period; and (iii) no thyroid therapy given during the study period.

We were given information about thyroid treatment on all the women who gave birth during the study period, including the entire study period. for example, a woman giving birth in 2011 also could have information on thyroid treatment from 2004 and up to 2018. For each pregnancy, we calculated three time points based on the ultrasound due date: (i) within 1 year before pregnancy, (ii) during pregnancy, or (iii) within 1 year after pregnancy. Women who redeemed a prescription for thyroid treatment from the pharmacy during the study period (2004–2018) were considered to undergo thyroid treatment and this was separately assessed for the three time points. Therefore, one woman can be represented as a case in each of the three time points.

### Statistics

The data were analyzed using STATA 16.1 software (STATA, College Station, TX, United States: StataCorp, 2019). Descriptive data were presented as numbers and percentages. Maternal age was presented as mean age of each year of due date with s.d. We calculated the percentage of women using either (i) thyroid hormone replacement therapy or (ii) antithyroid medication for each year in the three different time points. We explored the association between the use of thyroid therapy at the three different time points and year of due date using logistic regression and also adjusting for maternal age. We also adjusted the standard errors for clustering due to repeated pregnancies by calculating robust standard errors. Furthermore, we made prediction plots of the same association using the twoway fpfit command in Stata. These displays the predicted increase in thyroid therapy in the three different time points with 95% CIs.

### Ethical considerations

The study was approved by the Norwegian Regional Committees for Medical Health Research Ethics, REC South East C with reference number 95724.

Using data from the MBRN and NorPD in research are regulated by the Personal Health Data Filing System Act (Helseregisterloven). It is not necessary to obtain informed consent from the participants, as their personal identification numbers are deidentified.

## Results

**Figure 1:**
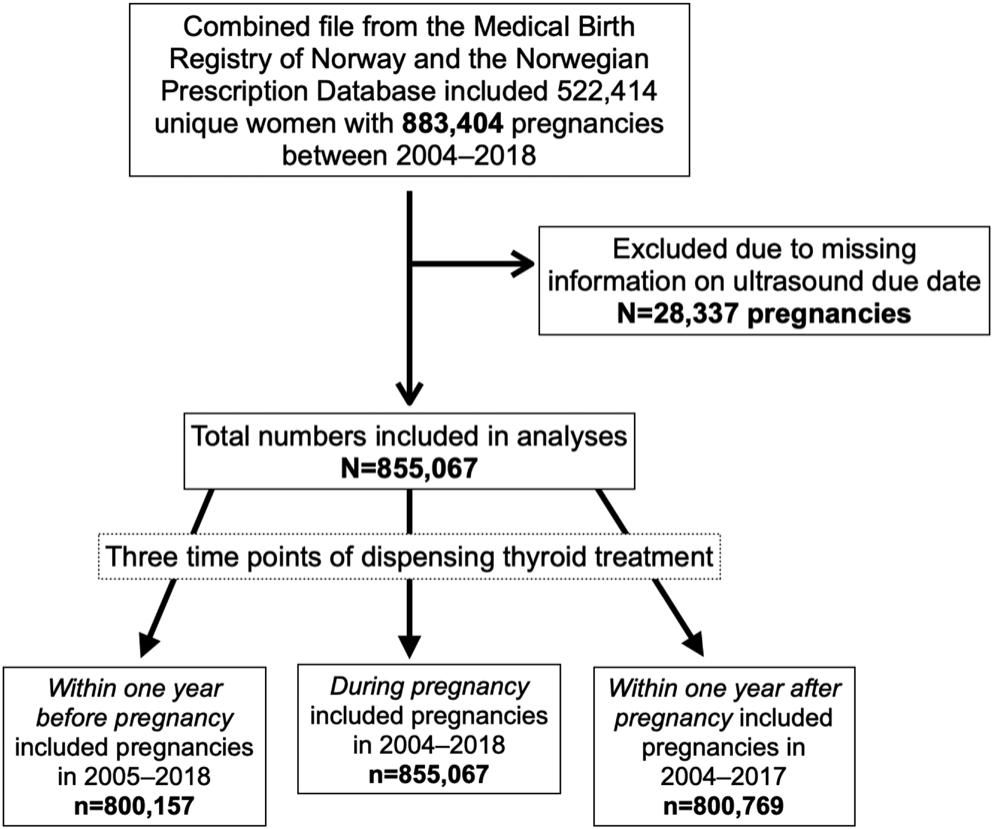
Flowchart displaying study population.

The datafile included 522,414 women with 883,404 pregnancies over the study period (Fig. 1). We used the ultrasound due year registered in the MBRN as a proxy for year of birth. This variable was missing for 28,337 pregnancies (3.2%). The number of pregnancies each year varied from 54,292 in 2018 to 59,307 in 2010. During 2004–2018, the mean maternal age increased from 29.6 to 30.5 years (Table 1).

**Table 1:** Description of study participants; *n* = 855,067.

Year of due date	Number of pregnancies	Mean maternal age (s.d.)
2004	54,910	29.6 (5.0)
2005	54,798	29.7 (5.1)
2006	56,135	29.7 (5.1)
2007	55,250	29.8 (5.2)
2008	58,314	29.7 (5.3)
2009	59,273	29.7 (5.3)
2010	59,307	29.8 (5.3)
2011	58,568	29.8 (5.3)
2012	58,399	29.9 (5.2)
2013	57,258	29.9 (5.2)
2014	57,245	30.1 (5.1)
2015	57,310	30.2 (5.1)
2016	58,066	30.3 (5.0)
2017	55,936	30.4 (4.9)
2018	54,298	30.5 (4.9)

During the study period, there was an increase in the proportion of women using thyroid hormone replacement therapy before, during, and within 1 year after pregnancy (Table 2).

**Table 2:** Frequency of thyroid hormone replacement therapy during study period by year of due date; *n* = 855,067.

Year of due date	Within 1 year before pregnancy		During pregnancy		Within 1 year after pregnancy		Total
	*n*	%	*n*	%	*n*	%	*n*
2004	–	–	800	1.46	1213	2.21	54,910
2005	924	1.69	1082	1.97	1316	2.40	54,798
2006	1108	1.97	1160	2.07	1371	2.44	56,135
2007	1135	2.05	1205	2.18	1505	2.72	55,250
2008	1234	2.12	1358	2.33	1611	2.76	58,314
2009	1297	2.19	1404	2.37	1660	2.80	59,273
2010	1437	2.42	1594	2.69	1804	3.04	59,307
2011	1363	2.33	1513	2.58	1737	2.97	58,568
2012	1463	2.51	1650	2.83	1922	3.29	58,399
2013	1454	2.54	1692	2.96	1957	3.42	57,258
2014	1508	2.63	1727	3.02	1941	3.39	57,245
2015	1605	2.80	1808	3.15	2002	3.49	57,310
2016	1689	2.91	1959	3.37	2167	3.73	58,066
2017	1609	2.88	1850	3.31	2063	3.69	55,936
2018	1650	3.04	1940	3.57	–	–	54,298
Total cases	19,476		22,742		24,269		66,487^a^

^a^Number of cases in total.

The largest increase in use was among pregnant women where the proportion increased 2.4-fold from 1.46% (*n*  = 800) in 2004 to 3.57% (*n*  = 1940) in 2018.

There was also an increase in the proportion of women using antithyroid medications before, during, and within 1 year after pregnancy (Table 3). The highest increase was among women during pregnancy which increased from 0.04 to 0.10%, a 2.5-fold increase.

**Table 3:** Frequency of antithyroid medication during study period by year of due date; *n* = 855,067.

Year of due date	Within 1 year before pregnancy		During pregnancy		Within 1 year after pregnancy		Total
	*n*	%	*n*	%	*n*	%	*n*
2004	–	–	20	0.04	88	0.16	54,910
2005	59	0.11	49	0.09	127	0.23	54,798
2006	67	0.12	44	0.08	108	0.19	56,135
2007	81	0.15	46	0.08	111	0.20	55,250
2008	80	0.14	41	0.07	122	0.21	58,314
2009	93	0.16	35	0.06	127	0.21	59,273
2010	69	0.12	35	0.06	122	0.21	59,307
2011	86	0.15	61	0.10	143	0.24	58,568
2012	74	0.13	49	0.08	163	0.28	58,399
2013	91	0.16	60	0.10	152	0.27	57,258
2014	81	0.14	44	0.08	136	0.24	57,245
2015	101	0.18	69	0.12	159	0.28	57,310
2016	103	0.18	68	0.12	184	0.32	58,066
2017	110	0.20	64	0.11	178	0.32	55,936
2018	94	0.17	56	0.10	–	–	54,298
Total cases	1189		741		1920		3850^a^

^a^Number of cases in total.

When adjusting for maternal age, the odds for using thyroid hormone replacement therapy during pregnancy increased by an average of 5% per year (odds ratio (OR): 1.05, 95% CI: 1.05–1.05; Table 4). The odds for using antithyroid medications during pregnancy increased by an average of 4% per year (OR: 1.04, 95% CI: 1.03–1.06).

**Table 4:** The association between thyroid therapy and year of due date. Crude and adjusted odds ratio (95% CI); *n* = 855,067.

	Crude	Adjusted^a^
Thyroid hormone replacement therapy		
Within 1 year before pregnancy^b^ (*n* = 19,476)	1.04 (1.04–1.04)	1.04 (1.03–1.04)
During pregnancy (*n* = 22,742)	1.05 (1.05–1.06)	1.05 (1.05–1.05)
Within 1 year after pregnancy^c^(*n* = 24,269)	1.04 (1.04–1.04)	1.04 (1.03–1.04)
Antithyroid medication		
Within 1 year before pregnancy^b^ (*n* = 1,189)	1.04 (1.02–1.05)	1.03 (1.02–1.05)
During pregnancy (*n* = 741)	1.05 (1.03–1.07)	1.04 (1.03–1.06)
Within 1 year after pregnancy^c^ (*n* = 1920)	1.04 (1.03–1.06)	1.04 (1.03–1.05)

^a^Adjusted for maternal age; ^b^Includes data from 2005 to 2018 only, *n* = 800,157; ^c^Includes data from 2004 to 2017 only, *n* = 800,769.

**Figure 2:**
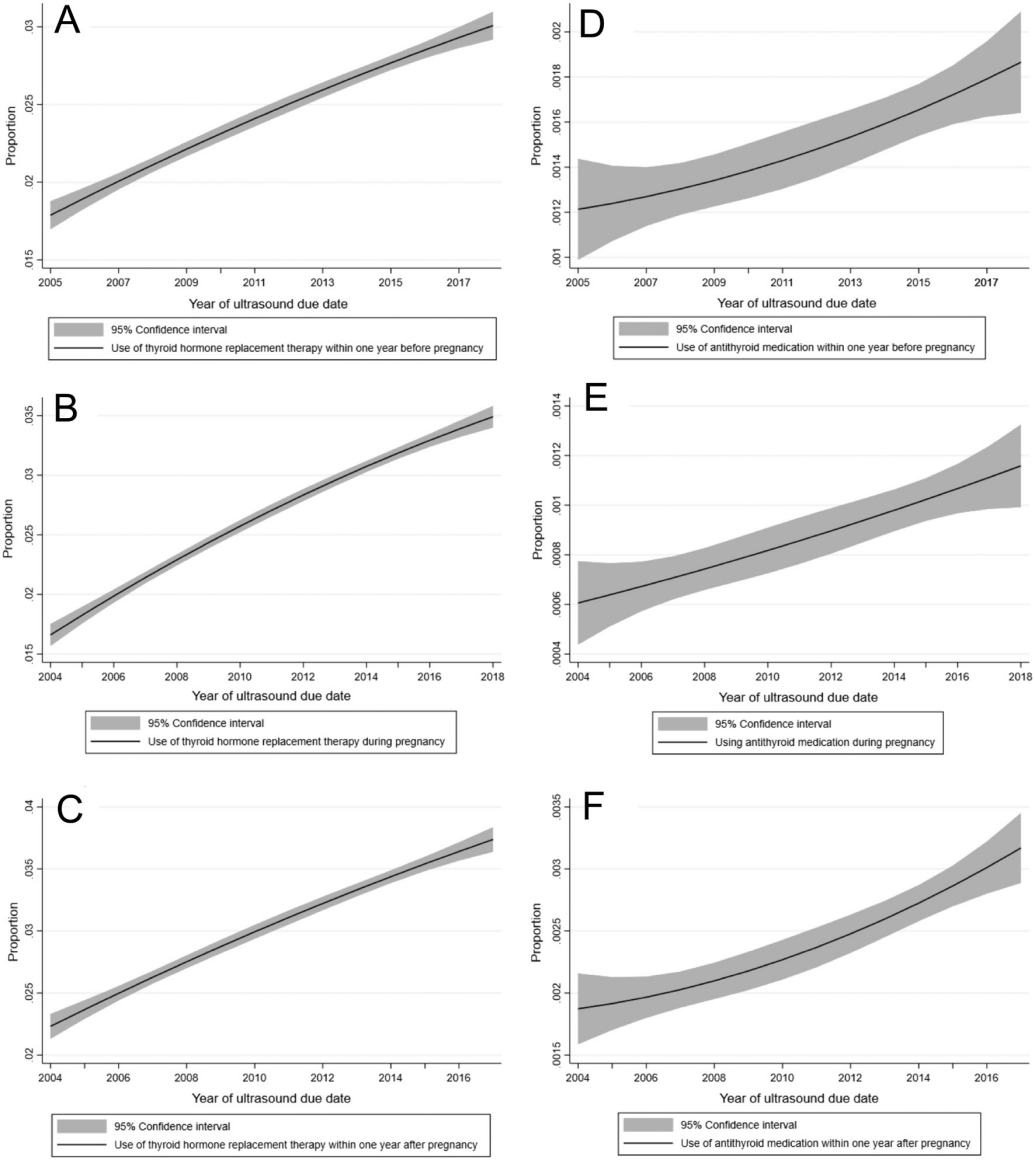
Prediction plots of proportion of women using antithyroid medication (D) within one year before pregnancy (including data from 2005–2018, *n* = 800,157), (E) during pregnancy (including data from 2004–2018, *n* = 855,067), (F) therapy within one year after pregnancy (including data from 2004–2017, *n* = 800,769).

Figure 2 displays prediction plots of proportion of women using thyroid hormone replacement therapy in the three different time points and proportion of women using antithyroid medication. They all predict an increasing trend.

## Discussion

This is the second study that quantifies the change in the number of prescriptions for thyroid medication around and during pregnancy on a national level throughout an extended time period, spanning various (inter)national guidelines. Using data from 855,067 pregnancies in Norway, we found a substantial increase in pregnant women using thyroid hormone replacement therapy during 2004–2018. We found that the odds for using thyroid hormone replacement therapy and antithyroid medication during pregnancy increased by an average of 5 and 4% per year, respectively.

Our results are very much comparable to the recent similar study in Finland which found a five-fold increase in levothyroxine use among pregnant women from 1.1% in 2004 to 6.2% in 2016 (14). We found a smaller increase in the Norwegian numbers (2.4-fold); however, the proportion of women using thyroid hormone replacement therapy was lower (3.57% in 2018). Our results are also in line with studies that were performed in non-pregnant populations. A study from the US showed an increase in prescription of thyroid hormone therapy from 5.1% in 1999–2000 to 6.1% in 2011–2012 (15). For the female population, the prescriptions increased from 8.0 to 9.3%, while for the age group 18–39 years (both genders), the increase was from 1.4 to 1.6% (15). In England, the number of prescriptions dispensed for thyroid hormone replacement therapy has more than doubled between 1998 and 2007 (16). A population study in Tayside, Scotland, found that all cases of thyroid disease increased from 2.3 to 3.8% between 1994 and 2001 (17).

The reasons behind the increased use of thyroid therapy could be many. First, an enhanced focus and better diagnostics could lead to an increased prevalence of thyroid disease. This is evident by the results of a repeated population-based cross-sectional study in Norway which found a strong decrease in proportion of cases with undiagnosed hypothyroidism from mid-1990s to late 2000s (18). Similar, a study from the US found that the proportion of treated women with subclinical hypothyroidism, retrospectively diagnosed in blood specimen, increased during their 5-year study period from 12 to 19% (5). However, one of the key clinical issues in this field is the definition of gestational thyroid disease (2). The most recent guidelines of the American Thyroid Association (ATA) from 2017 recommend calculating population-based trimester-specific reference ranges for serum thyroid-stimulating hormone (TSH) through assessment of local population data (2). Reference range determinations should only include otherwise healthy reference subjects that are thyroid peroxidase antibodies-negative and free of pre-existing thyroid disease, with an optimal iodine intake. However, most hospitals do not have such reference ranges available, which considerably limits the accuracy of clinical diagnoses. If calculation of hospital-specific reference ranges is not feasible, the ATA guidelines recommend using a fixed upper TSH reference limit of 4.0 mU/L, but no reference values for free thyroxine can be provided, making overdiagnosis an imminent problem (19). Establishing reference ranges for a Norwegian population could improve the diagnosis of gestational thyroid disease nationwide.

A second reason could be the increase in inadequate iodine intake among this group of the population (20, 21, 22). Since iodine cannot be synthesized, everyday diet may not guarantee optimal state of health (23). A recent observational study from Norway found that pregnant and postpartum women with mild-to-moderate iodine deficiency had altered thyroid function (24). However, they found no clear association between iodine status and thyroid dysfunction. New recommendations for iodine supplementations during pregnancy were introduced in 2017 and perhaps this will in the future reduce the proportion of fertile women in need of thyroid therapy (https://www.helsenorge.no/kosthold-og-ernaring/sma-grep-for-et-sunt-kosthold/derfor-trenger-vi-jod).

Although increasing iodine in diet may reduce the use of thyroid therapy in the pregnant population, endocrine disruptors could inhibit the uptake of iodine in the thyroid gland. Furthermore, experimental and epidemiological studies have shown that a wide spectrum of environmental contaminants have the potential to adversely affect the hypothalamic–pituitary–thyroid axis (9, 25). There has been an increasing concern that exposure to environmental contaminants during pregnancy may result in reduced maternal thyroid hormone synthesis affecting fetal neurodevelopment (26, 27). Further studies are needed to explore the exposure of these contaminants and to understand the extent of their role in the increased proportion of pregnant women using thyroid therapy in Norway.

Thirdly, increasing BMI among pregnant women has been observed, both in Norway and worldwide (28, 29). A positive correlation between TSH and BMI has been shown, and the relationship seems to be complex (30). Thyroid hormones participate in adipocyte differentiation and lipolysis regulation and adipocyte cytokines interact with regulation of thyrotropin (31). Unfortunately, the MBRN does not have good quality data on maternal BMI, and therefore, we could not explore the contribution of this variable in our study.

Increasing maternal age may lead to an increase in morbidity among the pregnant population (31). A population-based study from the US, using the National Health and Nutrition Examination Survey (NHANES III), found an association between elevated serum TSH with increasing age among women and men (32). Albeit this was only significant for the older age groups (40 years and older). Furthermore, a cohort study in China showed that among women delivered by cesarean section and those 35 years and older had lower free triiodothyroinine in cord blood compared to those under 30 years of age (33). In the current study, the mean maternal age increased by 0.9 years during the study period, and a sub-analysis showed an unadjusted correlation between thyroid medication use and increased age (*P* ≤ 0.001). However, as the mean increase in age is small, this is not a reason for concern related to our results.

Another possible contributor to the increased use of thyroid therapy could be that euthyroid women with thyroid autoantibodies under assisted reproductive therapy use thyroid hormone treatment. The proportion of babies born after assisted reproductive therapy has increased by 2.2% during the studied period (http://statistikkbank.fhi.no/mfr/).

In addition to all the possible reasons for the increase in pregnant women using thyroid hormone replacement therapy, we also need to address the possible consequences this may have for women and their infants. Both too high and too low maternal free thyroxine concentrations are associated with 1.4–3.8 points reduction in child IQ (6). To reduce possible consequences, further studies are needed as current evidence is unconclusive regarding screening and treatment during pregnancy (2).

A major strength of our study is the use of MBRN and NorPD as source of data. In 2020, the proportion of invalid or missing National ID numbers registered to a prescription was 0.1% (http://www.norpd.no/). Data collected from MBRN and NorPD gives us a comprehensive overview of pregnant women and new mothers using thyroid therapy. One of the disadvantages of our study is the use of ultrasound due date as a proxy for year of birth, and not the actual birthday. This could lead to some misclassifications of cases in year of birth as some of them would have given birth pre- or post-term. However, this is not differential and would not affect our results significantly. A second disadvantage in our data is the assumption that prescriptions for medication with ATC code H03BA and H03AA are used to treat diagnosed hyperthyroidism and hypothyroidism. There is a risk that some women have been given these medications without a proper diagnosis. Furthermore, the shortfall of data on other possible confounding factors limited our opportunity to explore other possible determinants for our result.

## Conclusion

During the last 15 years, there has been a substantial increase in the use of thyroid hormone therapy medication in Norwegian pregnant women. We speculate that this could be due to an increased awareness in combination with overdiagnosis because of inappropriate diagnostic criteria. To truly understand the possible causes and consequences of this development, further research is warranted.

## Declaration of interest

The authors declare that there is no conflict of interest that could be perceived as prejudicing the impartiality of the research reported.

## Funding

This work was supported by Innlandet Hospital Trust Research Fund (grant number 150424).

## Author contribution statement

All authors have contributed substantially to the work reported in this paper. Conceptualization, K S B; methodology, T A S and K S B; data collection, K S B; statistical analysis, K S B and T A S; writing – original draft preparation, K S B and K R; writing – review and editing, T A S, T I M K, B G N, K R and K S B; visualization, T A S and K S B; supervision, T A S and T I M K; project administration, K S B; funding acquisition, K S B.
